# The Role of School Environment on the Sustainable Development of Pre-Schoolers’ Motor Creativity

**DOI:** 10.3390/sports13070229

**Published:** 2025-07-11

**Authors:** Despoina Ourda, Anna Kavoukoglou, Athanasios Gregoriadis, Vassilis Barkoukis

**Affiliations:** 1 Department of Physical Education and Sport Science, Aristotle University of Thessaloniki, 54124 Thessaloniki, Greece; kavoua@gmail.com (A.K.); bark@phed.auth.gr (V.B.); 2 Department of Early Childhood Education, Aristotle University of Thessaloniki, 54636 Thessaloniki, Greece

**Keywords:** preschool children, creativity, movement, teachers, infrastructures

## Abstract

This study examined the influence of student–teacher relationships and school infrastructure on preschool children’s motor creativity, encompassing fluency, originality, and imagination. Twenty teachers completed the Student–Teacher Relationship Scale for 200 children (10 children per teacher). The research team recorded aspects of the school’s physical environment through Movement Play Scale and assessed children’s motor creativity via the Thinking Creatively in Action and Movement test. The results revealed that dimensions of the student–teacher relationship, such as conflict and dependency, negatively impacted fluency, and originality components of motor creativity. Contrary to expectations, teacher participation in movement activities did not significantly contribute to motor creativity, potentially due to over-direction limiting children’s autonomy. Similarly, the school’s infrastructures were negatively linked to fluency and originality. The findings underscore the importance of nurturing autonomy-supportive environments and balancing guidance with opportunities for independent exploration. Teachers should also invest in adaptable educational spaces to foster creativity without encouraging dependency. This study emphasizes the critical role of supportive relational and environmental factors in shaping preschool children’s creative movement abilities.

## 1. Introduction

Kindergarten serves as the initial educational institution for the majority of children, marking the commencement of obligatory education. Kindergarten is widely recognized as a form of early childhood education that serves as a preparatory phase before formal schooling. It serves as the setting for the cultivation of psychological, cognitive, and interpersonal growth [1]. Consequently, it is imperative for the school environment to provide assistance and facilitate the progress of children’s learning and development, while also promoting their playful behavior, inventiveness, and mobility. Importantly, motor creativity is a crucial aspect of early childhood development, as it significantly contributes to children’s physical, cognitive, and social development [2,3,4]. The attainment of these objectives can be facilitated by engaging in meaningful interactions that enhance learning through play and provide positive reinforcement for students’ endeavors [5].

### 1.1. Physical Movement in Preschool Education and Motor Creativity

Incorporating movement-based activities into the daily curriculum and providing increased opportunities for physical activity during early childhood foster a natural inclination toward higher physical activity levels among preschool-aged children, especially when facilitated through play [6,7]. Through movement-based activities, children explore spatial and temporal concepts, enhancing their responsiveness and overall capabilities [8]. The physical education lesson, in particular, is vital in nurturing young individuals’ abilities to question, solve problems, interact, express thoughts, listen actively, foster creativity, and communicate effectively [9]. Notably, children in schools with organized physical activity programs and dedicated spaces are more active compared to those in schools that neglect physical education and lack appropriate facilities [10]. The development of motor creativity, encompassing the ability to produce novel and adaptable movement patterns, is a core objective of physical education programs in preschool education [11]. Motor creativity refers to the capacity to generate novel and effective movement patterns in response to specific tasks or challenges, distinguishing it from general creativity, which encompasses the ability to produce original and valuable ideas across various domains. Understanding motor creativity is crucial, as it plays a significant role in enhancing motor competence and adaptability. Research indicates that early development of motor creativity in children has been linked to long-term benefits, including better problem-solving skills and overall cognitive development [12]. Three key dimensions of motor creativity have been identified; fluency, imagination, and originality [13,14]. Fluency refers to the quantity of different movement solutions a person can generate in response to a given task and emphasizes the ability to produce multiple motor responses rather than just a single one. Imagination represents the ability to generate diverse and flexible motor solutions that deviate from conventional or routine movements, and it reflects the capacity to adapt movements creatively. Originality highlights the uniqueness of the generated movements and involves producing motor responses that are rare or uncommon within a given context. These three constructs collectively provide a comprehensive framework for evaluating motor creativity because they cover the breadth, adaptability, and novelty of movement solutions [13,14,15]. A highly creative mover is not just someone who generates many solutions (fluency) but also someone who thinks beyond conventional movement patterns (imagination) and produces movements that stand out in their uniqueness (originality). By understanding and studying all three dimensions, researchers and practitioners can obtain a well-rounded picture of motor creativity [14].

Motor creativity is a fundamental aspect of early childhood development. It enables children to explore their physical environment, express emotions, and solve problems through movement [10]. Engaging in creative movement activities enhances motor skills [16]. Evidence further links motor creativity to cognitive, social, and emotional development. In particular, creative movement encourages children to experiment with new ideas, collaborate with peers, and express themselves in non-verbal ways, thus enhancing their social skills and self-confidence [17,18]. Furthermore, it supports cognitive processes such as problem-solving and critical thinking, as children navigate spatial and temporal challenges inherent in creative movement tasks [19,20].

Research indicates that interventions focusing on motor creativity can lead to improvements in children’s overall development, including their knowledge about health and attitudes towards healthy behaviors [2,21]. Early development of motor creativity has been associated with increased physical activity levels and overall well-being, laying the foundation for a healthy and active lifestyle in adulthood [22,23].

Preschool education offers a unique opportunity to nurture motor creativity. This critical stage of development serves as a platform for children to engage in creative play and motor exploration, both of which are essential for their holistic growth [3,4]. Research highlights the role of structured interventions and enriched environments in fostering motor creativity, suggesting that children provided with diverse physical stimuli exhibit greater fluency, originality, and imagination in their movements [11,22,23,24,25]. Thus, the role of the school environment, including the quality of space, resources, and teacher–student interactions, is pivotal in shaping motor creativity. Studies indicate that well-designed physical spaces equipped with age-appropriate resources provide children with opportunities to engage in spontaneous, unstructured movement, a key driver of motor creativity [26,27]. Similarly, positive and supportive teacher–student relationships have been shown to encourage exploration, autonomy, and creativity in young learners [28].

### 1.2. Student–Teacher Relationships

The relationship between children and teachers is a critical factor in shaping preschool children’s motor, social, and psychological development [29,30]. Research has demonstrated that positive and supportive teacher–student relationships foster an environment of trust and emotional security, which is essential for children to explore and engage in new activities, including those that enhance motor skills [31,32]. According to self-determination theory, teachers who provide encouragement and constructive feedback create a safe space for children to experiment with creative movements, which in turn supports motor development and builds self-confidence [28,33].

From a cognitive and social development perspective, strong student–teacher relationships promote an adaptive growth [34,35]. According to self-determination theory, teachers should target competence through encouragement and feedback, and nurture children’s autonomy, in order to foster their intrinsic motivation and self-regulation. Heightened intrinsic motivation and self-regulation helps children learn to navigate social dynamics and build interpersonal skills, reduce behavioral challenges, and focus on creative and constructive activities [36], such as movement-based tasks. Furthermore, studies have shown that preschoolers in classrooms with high teacher–student closeness demonstrate better peer interactions and are more likely to participate in cooperative play, a key context for developing social behaviors [37,38,39,40].

Teacher–student relationships also influence children’s emotional well-being and resilience. Emotional support by teachers helps children establish fundamental social and emotional skills such as communication and relationship skills, responsible decision-making, social awareness, and self-awareness [41]. Teachers who create a nurturing atmosphere encourage children to take risks, explore novel ideas, and express themselves through movement, thereby supporting the psychological foundations of creativity [42].

The link between student–teacher relationships and motor creativity is less explored despite studies highlighting the role of teacher support in fostering creative expression [17]. Moreover, teachers who actively participate in or facilitate movement-based tasks can directly influence children’s creative output by offering guidance and inspiration [23]. For example, structured programs led by supportive teachers have been shown to enhance fluency and originality in preschoolers’ creative movement [11].

### 1.3. Physical Environment

Kindergarten represents the first formal educational setting where children transition from the nurturing environment of their family, often accompanied by feelings of uneasiness and fear [43]. A well-designed physical environment, such as open play spaces, diverse equipment, and opportunities for unstructured physical activity, provides children and athletes with the freedom to explore and develop novel movement patterns. Research suggests that enriched environments with varying stimuli enhance divergent thinking in motor tasks, leading to higher motor creativity [44,45]. Therefore, the design of the kindergarten environment should foster a positive predisposition in children. An ideal setting is welcoming, close-knit, and cozy, incorporating decorative elements and appropriate equipment to create a secure atmosphere that nurtures trust [26]. Kindergartens should provide stimuli that encourage engagement and support cognitive development, as the physical environment significantly influences children’s cognitive and physical activities [27]. Spatial constraints, however, can negatively affect various aspects of development and functioning [46]. Research suggests that thoughtfully arranged environments promote engagement and discovery among preschool children [47]. Flexibility is a crucial characteristic of functional educational spaces, enabling transformations without structural modifications to accommodate diverse activities [46,48]. This flexibility and adaptability are particularly beneficial in compact spaces, allowing for a wider range of activities without spatial limitations [49]. Promoting physical activity requires creating an environment that supports and enhances children’s motor development. Boon et al. [50] emphasize the importance of spatial design and appropriate equipment in facilitating motor play, which is integral to the development of physical skills. Such environments should prioritize comfort, safety, and adaptability, allowing children to engage confidently. Additionally, providing diverse, appropriately sized equipment—such as balls, hoops, bars, and balance beams, tailored to children’s abilities and developmental needs is essential. Kindergarten settings, in particular, should incorporate flexibility to allow modifications that optimize developmental outcomes [51]. However, evidence suggests that kindergarten settings often lack structured and open spaces for children to engage in spontaneous and unrestricted movement. Kouli et al. [52] found that in Greece, the quality of physical environments and infrastructure was assessed as ranging from low to moderate. This underscores the critical need to more thoroughly study the role of physical environments, as they may play a significant role in influencing children’s participation in physical activity.

### 1.4. The Present Study

Teacher–student relationships and the physical environment play a crucial role in predicting and fostering motor creativity by encouraging autonomy, exploration, and exposure to diverse movement opportunities [32,53]. Overall, supportive school environments improve children’s creativity, self-confidence, and imagination [17]. Motor creativity is a foundational aspect of early childhood development, integrating physical, cognitive, and emotional growth [2,17,20,21,22,54]. Despite its importance, research indicates that many preschool environments do not adequately foster motor creativity, often prioritizing structured motor skills over exploratory movement [22]. This is concerning, as low levels of motor creativity in early childhood have been linked to reduced problem-solving abilities, lower engagement in physical activity, and hindered social interaction skills [15,23]. Recent studies suggest that structured interventions can significantly enhance motor creativity, leading to improvements in both cognitive and physical outcomes [25]. For example, children exposed to movement improvisation and open-ended play environments demonstrate 25–40% higher scores in creativity-related motor tasks compared to those in rigidly structured programs [22]. Additionally, longitudinal data show that early motor creativity positively predicts physical activity participation in later childhood and adolescence, underscoring its role in long-term health and well-being [54]. Given these findings, it is imperative to examine how school environments, including teacher behaviors and physical infrastructure, influence motor creativity. In particular, there are no studies investigating the effect of aspects of teacher–student relationships such as conflicts, student dependency, and level of close relationships. Accordingly, with respect to the physical environment of the school, the existing literature has focused on the provision of opportunities through educational programs but did not adequately investigate the role of physical resources and environment in the promotion of motor creativity. Despite its critical importance, research on the determinants of motor creativity in preschool settings remains limited, particularly concerning the interplay between teacher behaviors and physical environments. This gap is significant because early childhood is a sensitive period for fostering creative abilities through movement, which supports lifelong skills such as problem-solving, social interaction, and emotional regulation. Previous studies in Greece have emphasized the role of structured physical education in enhancing motor creativity [11,25], yet they often neglect the nuanced contributions of teacher–student interactions and the physical environment. Moreover, previous research focused on older children [55], leaving a critical knowledge gap in understanding how preschool environments shape the development of motor creativity, and motor development overall. To address this gap, the present study was designed to investigate how the school environment, including teacher-related factors and school infrastructures, influence motor creativity in preschool children. Based on the existing literature, we hypothesized that (a) enhanced physical environment positively influences children’s motor creativity, and (b) positive student–teacher interactions are positively associated with children’s motor creativity.

## 2. Materials and Methods

### 2.1. Sample

Data from 200 pre-school children (100 girls and 100 boys) attending 20 different private and public kindergartens in Thessaloniki, Northern Greece, was collected in the study. All children were preschoolers and toddlers with an age range from 49 to 72 months (M = 61.40 months, SD = 7.30). The majority of the children were of Greek nationality, but there were 16 children of different nationalities (7 Albanian, 3 Georgian, 4 German and 2 Dutch). The teacher was the unit of selection. Following an open invitation to social media and contacts in kindergartner teacher networks, 20 teachers responded in the predetermined time and were included in the study. Each teacher was teaching in a separate classroom with an average size of 21 children. Participants were required to meet the following conditions to ensure consistency in teaching experience and familiarity with the children (a) minimum of five years of teaching experience in early childhood education, ensuring that all teachers had a well-established understanding of student behavior, motor development, and classroom dynamics, (b) continuous employment at the same school since the beginning of the academic year, allowing for sustained teacher–student interactions and minimizing variability due to teacher turnover, and (c) full-time employment, ensuring that teachers were regularly engaged with children and had comprehensive insight into their motor and social development. The exclusion criteria employed to eliminate potential confounding variables that could influence teacher perceptions and student outcomes included (a) participation in professional development programs focused on physical education or creativity within the past two years, as this could have introduced biases in how teachers facilitate movement-based activities and assess creativity, (b) teachers with limited engagement in direct classroom instruction, such as substitute or part-time teachers, were excluded to ensure consistent teacher–student relationships across the sample, and (c) schools implementing specialized motor creativity interventions or experimental movement-based curricula were excluded to avoid external influences on children’s creative movement outcomes. A post hoc power analysis using GPower confirmed that a sample of 200 provides sufficient statistical power (>0.80) to detect medium-sized effects (f^2^ = 0.15) in regression analyses, ensuring the robustness of our findings.

### 2.2. Measures

Student–teacher relationship: The Student–Teacher Relationship Scale (STRS) [28] was used to assess the relationship between teacher and child. It is used for children aged 4–8 years old [28]. The scale consists of 28 items, which are answered by the teacher and measure three dimensions of relationship; close relationship (11 items, e.g., ‘I share an affectionate, warm relationship with this child’), conflict (12 items, e.g., ‘This child easily becomes angry at me’), and dependency relationship (5 items, e.g., ‘This child is overly dependent on me’). Teachers responded to each item on a 5-point Likert scale ranging from 1 (not applicable) to 5 (absolutely applies).

Physical environment: The instrument used in the current study was the Movement Play Scale (MPS) [56,57], which consists of three items, each accompanied by detailed explanatory notes:

Item 1—Space and Resources: This item includes 11 indicators that include the evaluation of whether the range of activities, resources, and the environment allows children to engage spontaneously in movement activities alone, with peers, or with adults. The indicators also include an estimation of whether there is a wide variety of equipment and resources that are easily accessible for children to use as needed, both indoors and outdoors, as well as adequate safety measures.

Item 2—Adults Engaging in Movement with Children: Comprising 10 indicators, this item assesses whether children are encouraged to express themselves freely through movement. In addition, the indicators assess the quality of interaction and inclusivity, and movement diversity. The indicators also examine opportunities provided to parents to enhance their understanding of children’s movement and whether staff expand their knowledge through additional training, reading, or professional development courses.

Item 3—Planning for Movement-Play from Observations of Children: This item includes 9 indicators that assess whether trained staff plan specific movement activities based on observations of children’s individual interests and needs, both indoors and outdoors. It also evaluates whether parents’ observations are incorporated into planning and assessments, and whether other professionals collaborate with staff in planning and contributing to children’s movement activities.

Members of the research team assessed each indicator. The criteria were evaluated on 7-point scale from 1 (inadequate) to 7 (excellent). The MPS has been validated in Greek environments and is considered a valid instrument for the evaluation of the role of space, resources, adult involvement, and planning in fostering movement play among preschool children [52].

Motor creativity: The version of the Thinking Creatively in Action and Movement test (TCAM) adapted to preschoolers was used to assess motor creativity [15]. This test was designed to measure three dimensions of creative movement, fluency, originality, and imagination. The test includes four activities that provide evidence of the main methods that children use to express their creative abilities. The first activity aims to identify children’s ability to generate alternative ways of moving. The activity has the stem question “In how many ways?“ and the observer asks the children to think of how many ways they can move from one point to another, which are defined by strips of tape placed on the ground. Both verbal and motor answers and a combination of both are acceptable. The researcher motivates children, allows them to express themselves and records the ways in which they perform. Fluency and originality are assessed with this activity.

The second activity aims to assess children’s ability to imagine, pretend, be in a situation and play roles. The activity has the stem question “Can you move like?“ and includes six different tests—situations where children are asked to imitate them through movement. Specifically, the researcher asks the children to (1) imagine that they are a tree and that there is a very strong wind blowing and show how the tree would move, (2) imagine that they are a rabbit and someone is chasing it and show how it would jump, (3) show how they would swim if they were fish, (4) show how they would crawl on the ground if they were snakes, (5) imagine they were driving a car on a long road, and (6) push an imaginary elephant sitting on something of their own. The researcher is asked to circle the appropriate level of imitation-response on a Likert scale of 1–5, where 1 = no movement, imitation, 3 = adequate performance, and 5 = excellent, perfect representation.

The third activity has the stem question “In what other ways?” and aims to identify whether children can invent unusual and original ways of performing a movement beyond the standard ones. The researcher gives children a plastic cup and asks them to show how many ways they can put it in a basket. The researcher records the children’s motor and verbal responses and scores them according to their originality and fluency.

The fourth activity has the stem question “What else could it be?” and measures children’s fluency and originality when they are asked to imagine that the plastic cup becomes a toy object. Children are then asked to play with the cup when it ceases to have its normal use. The glass in the children’s imagination can become a bracelet, a pot, a hat or even a plate for their pet’s food.

TCAM is completed by an observer, member of the research team. The answers given by the child in each of these activities are scored according to the level of originality and then a set is completed, which identifies the 3 scales of measurement, which are fluency, total number of answers, originality, number of different answers and imagination, rarity of answers [58]. In the present study, children’s responses were recorded by two researchers. The inter-rater agreement was 83%.

### 2.3. Procedure

Permission was obtained by the school’s authority and the teachers. Children’s parents were informed by the school authority about the aims and content of the test and gave their consent for their children to participate. In this study, we recruited 20 kindergarten teachers who voluntarily agreed to participate. Each teacher provided data on the student–teacher relationship by completing the Student–Teacher Relationship Scale (STRS) for 10 randomly selected children from their class, ensuring a diverse and representative sample. Following this, the research team conducted direct classroom observations and completed the Movement Play Scale (MPS), assessing the physical environment, teacher engagement, and structured movement activities. This step allowed for an objective evaluation of the learning space and teacher facilitation of movement-based interactions. The MPS was completed for each classroom, in a teaching time of approximately 3–4 h, as the logistical infrastructure of the room and the participation of each teacher had to be completed. Finally, the research team individually assessed each child’s motor creativity using the Thinking Creatively in Action and Movement (TCAM) test. This test was administered one-on-one, ensuring that each child’s fluency, originality, and imagination in movement-based tasks were accurately recorded without external influences from peers or teachers. Completion of TCAM lasted 30 min for each child. Both school teachers and parents were reassured about the anonymity of responses and the confidential treatment of the data. To protect anonymity, the scores of the children across the measures were matched based on a code that the teacher assigned to each child.

Data analysis: The statistical package IBM SPSS Statistics for Windows (Version 28.0; IBM Corp., Armonk, NY, USA) was used to analyze the results. Descriptive statistics were performed. Cronbach’s α coefficient was used to examine the internal consistency of the continuous variables under consideration. Pearson’s correlation index r was used to examine the associations among the variables. Regression analysis was used to examine the hypotheses. The significance level was set at 0.05.

## 3. Results

### 3.1. Descriptive Statistics

The mean age of the children was 61.40 months (SD = 7.30). The mean age of the boys was 61.84 months (SD = 7.55) and of the girls 61.05 months (SD = 7.05). Descriptive statistics of all variables are presented in Table 1. The analysis of correlations among the studies variables is reported in Table 2.

### 3.2. Effect of Space and Resources on Motor Creativity

With respect to the first hypothesis, the linear regression analysis provided significant insights into the effect of space and resources on motor creativity. For fluency, the model explained 46% of the variance (R^2^ = 0.46, F = 58.81, *p* < 0.001, Cohen’s f = 0.89), with the following significant predictors: space and resources (b = −0.13, t = −2.87, *p* < 0.05), teacher participation (b = −0.75, t = −10.14, *p* < 0.001), and planning (b = 0.19, t = 2.34, *p* = 0.02). For originality, the model accounted for 41% of the variance (R^2^ = 0.41, F = 48.40, *p* < 0.001, Cohen’s f = 0.72). Significant predictors included space and resources (b = −0.15, t = −2.29, *p* < 0.05), teacher participation (b = −0.69, t = −9.00, *p* < 0.001), and planning (b = 0.17, t = 2.09, *p* < 0.05). In contrast, for imagination, none of the variables tested in the model emerged as significant predictors (R^2^ = 0.00, F = 0.74, *p* > 0.05, Cohen’s f = 0.01).

### 3.3. Effect of Student–Teacher Relationship on Motor Creativity

With respect to the second hypothesis, the linear regression analysis provided information on the association of student–teacher relationship and motor creativity. For fluency, the model explained 19% of the variance (R^2^ = 0.19, F = 17.35, *p* < 0.001, Cohen’s f = 0.27), with conflict (b = −0.29, t = −3.80, *p* < 0.001) and dependency (b = −0.30, t = −2.98, *p* < 0.01) emerging as significant predictors. For originality, the model explained 19% of the variance (R^2^ = 0.19, F = 16.14, *p* < 0.001, Cohen’s f = 0.24), with conflict (b = −0.25, t = −3.32, *p* < 0.001) and dependency (b = −0.34, t = −3.40, *p* < 0.001) being the significant predictors. However, the results of the linear regression analysis indicated that none of the variables emerged as significant predictor of imagination (R^2^ = 0.02, F = 1.50, *p* > 0.05, Cohen’s f = 0.02).

## 4. Discussion

The primary aim of this study was to investigate how the school environment, including teacher-related factors and school infrastructures, influences motor creativity in preschool children. The results of the analyses indicated a negative association between conflict and dependency, as well as space and resources and teacher participation with motor creativity dimensions whereas a positive association emerged with planning.

More specifically, the hypothesis that positive student–teacher relationships will enhance motor creativity was partially supported. Regression analyses revealed that dimensions such as conflict and dependency negatively predicted fluency and originality, while imagination remained unaffected. These findings align with self-determination theory [46], which emphasizes that supportive relationships foster autonomy and intrinsic motivation, which are critical drivers of creative exploration. Supportive teacher–student relationships may create an environment that nurtures these needs, encouraging children to explore movement creatively without fear of judgment or constraint. In this respect, Ahmadi et al. [34] developed a classification system for need-supportive and motivational behaviors, which could serve as a strong foundation for designing intervention programs aimed at enhancing intrinsic motivation through movement activities. Conversely, dependency and conflict undermine the psychological safety required for experimentation, potentially inhibiting creative processes. As suggested by Spilt et al. [59] high levels of dependency were associated with lower levels of independent activities in the classroom. This evidence highlights the necessity of nurturing teacher behaviors that promote autonomy-supportive environments. Preschool children, due to their developmental stage, benefit greatly from interactions characterized by emotional warmth and constructive feedback. In this respect, teachers should emphasize fostering emotionally supportive relationships and minimizing dependency. These findings signify that autonomy plays a crucial role in educational settings by fostering an environment where children feel free to explore, experiment, and express creativity without fear of judgment or restriction [53]. Our findings contribute to self-determination theory by demonstrating that supportive student–teacher relationships enhance motor creativity through autonomy and intrinsic motivation in preschool education [53]. Given the limited studies on motivation in preschool education through the lens of self-determination theory, our study provides preliminary support to the tenets of the theory.

Contrary to expectations, teacher participation in movement activities was negatively associated with the motor creativity components. This result diverges from prior findings suggesting that active teacher involvement enhances children’s engagement and creative output [60]. One plausible explanation is the potential for over-direction or imitation when teachers participate, which might limit children’s autonomy in exploring novel movement patterns. This finding also supports the negative association of dependency with motor creative found in this study. If this is the case, high levels of teacher participation may result in higher dependency from the teacher, and subsequently, decreasing children’s autonomy or the challenge to create something new. This finding underscores the importance of balancing guidance with opportunities for independent experimentation. Thus, teachers need to adopt facilitative rather than directive roles during movement activities [34]. Nevertheless, future research could explore the nuances of this relationship, examining how varying degrees of teacher involvement influence motor creativity.

An interesting finding was the lack of significant predictive effects of student–teacher relationships and school infrastructure on imagination in motor creativity. This finding can be attributed to several theoretical and empirical factors. First, imagination in motor creativity is primarily an internal cognitive function, often driven by intrinsic motivation and personal cognitive flexibility rather than external environmental or relational factors [20]. Unlike fluency and originality, which can be shaped by teacher guidance, space availability, and structured movement activities, imagination may require deeper cognitive and affective engagement that is not necessarily influenced by external constraints. Research suggests that imagination is strongly linked to divergent thinking processes and internalized self-directed exploration, which may not be directly fostered by the physical environment or student–teacher interactions [44]. Additionally, while enriched environments have been shown to facilitate exploratory behaviors, they may not necessarily encourage abstract and spontaneous imaginative thought unless coupled with specific cognitive and emotional scaffolding [45].

Unexpectedly, the hypothesis that flexible and resource-rich school infrastructures positively influence motor creativity was not supported. Space and resources were negative predictors of fluency and originality. These findings are in contrast with earlier research indicating that enriched environments stimulate exploratory behaviors and novel movement patterns [26]. While previous research suggests that enriched environments foster creativity, we propose that overly structured settings may inadvertently limit children’s spontaneous movement exploration. This aligns with theories suggesting that creativity thrives in settings with moderate constraints, where children must actively generate novel solutions [60]. Additionally, high teacher involvement in structured play may reduce autonomy, leading to imitation rather than self-directed exploration [61]. Future research should further investigate the balance between structured resources and free play in promoting motor creativity in early childhood settings. Furthermore, the lack of effect on imagination might stem from its dependence on intrinsic cognitive factors rather than external stimuli. The absence of predictive effects in the regression analysis could also reflect the limitations of existing school environments in stimulating imagination beyond structured and observable movement activities. For example, teacher participation, which was negatively associated with fluency and originality, may inadvertently suppress children’s autonomous imaginative exploration by imposing predefined movement patterns [23]. Similarly, a well-structured physical environment may encourage movement diversity and motor skill development, but not necessarily the spontaneous, abstract, and flexible thinking required for imaginative motor responses [61,62]. Incorporating storytelling or imaginative scenarios into physical activities might better stimulate imagination. Theoretically, this suggests that while infrastructure plays a foundational role in facilitating creative movement, fostering imagination might require additional cognitive and emotional scaffolding. Practically, this calls for investments in adaptable and diverse physical environments that encourage spontaneous play. Schools should prioritize designs that maximize flexibility and provide a variety of movement materials. Overall, these findings highlight the need for further exploration of how internal cognitive processes, emotional states, and unstructured play opportunities contribute to the development of imagination in motor creativity. Future research should consider incorporating interventions that explicitly encourage free play, role-playing, and storytelling within movement-based activities to better support the development of motor imagination [12,63].

## 5. Limitations and Conclusions

Several limitations warrant consideration. First, the sample was not randomly selected and was recruited from a single geographical region, limiting the generalizability of findings. Participants were drawn from kindergartens willing to cooperate, potentially introducing selection bias. Second, the study did not control for external stimuli that children might have encountered outside the school setting, which could have influenced their motor creativity. Third, teacher-reported measures of student–teacher relationships may be subject to social desirability bias, potentially skewing the results. Also, the teacher–student relationship evaluation was based on self-reports provided by teachers; in future studies, independent observers could provide meaningful data that enhances our understanding of the effect of teacher–student relationships and school physical environment on children’s motor creativity. Lastly, the dimensions of fluency and originality are highly correlated, which implies multicollinearity. Although these constructs are conceptually different, they are measured with the same items of TCAM. Thus, future research should further investigate the structure of TCAM and its ability to distinctly measure fluency and originality. Overall, to address these limitations, future research should adopt longitudinal designs to capture developmental trajectories of motor creativity and account for the influence of external stimuli, utilize qualitative and mixed methods designs to obtain more thorough information and explore interventions that integrate emotional, cognitive, and environmental components. Nevertheless, this study underscores the multifaceted nature of motor creativity in preschool children, influenced by relational and environmental factors. The findings of this study have several practical implications for early childhood educators, school administrators, and policymakers. First, the negative association between structured school environments and motor creativity suggests that providing opportunities for unstructured movement play may be more beneficial than rigidly organized activities. Schools should design flexible learning spaces that encourage children to explore movement freely rather than prescribing specific actions. Second, the role of teacher involvement requires careful balancing. While teacher engagement is critical, excessive direction may reduce children’s autonomy in movement exploration, leading to lower creativity. Training programs should equip teachers with strategies that foster autonomy-supportive interactions rather than highly structured interventions. Third, given the significant influence of student–teacher relationships on motor creativity, schools should prioritize fostering emotionally supportive and autonomy-enhancing interactions. Providing professional development focused on minimizing dependency and conflict in teacher–student relationships may enhance creative movement development. Lastly, policymakers should consider revising early childhood curricula to emphasize open-ended movement activities. Ensuring access to versatile, adaptable physical spaces and materials can help balance structured learning with creative exploration, ultimately fostering children’s physical, cognitive, and social development. Overall, the study findings highlight the critical role of supportive teacher–student interactions to enhance creative movement. Our study revealed that the physical environment and resources significantly impact motor creativity. Limited space and equipment were linked to lower creativity levels, while balancing guidance and autonomy proved crucial for enhancing creative outcomes. Addressing these factors can help educators better support the holistic development of motor creativity in early childhood settings.

## Figures and Tables

**Table 1:** Descriptive statistics of the variables.

	Mean	SD	Skewness	Kurtosis	Cronbach α
Fluency	113.84	35.83	0.62	−0.87	-
Originality	112.33	27.88	0.77	−0.61	-
Imagination	96.33	10.77	−0.42	0.09	-
Infrastructure	4.40	1.35	−0.75	0.10	-
Participation	4.35	1.65	−0.30	−0.68	-
Planning	2.90	1.04	−0.06	−0.62	-
Close relation	3.19	0.71	−0.36	0.05	0.72
Conflict	1.86	0.65	0.94	0.13	0.86
Dependency	2.44	0.73	0.11	−0.41	0.83

**Table 2:** Analysis of correlation.

	1	2	3	4	5	6	7	8
1. Fluency	1							
2. Originality	−0.96 **	1						
3. Imagination	0.26 **	0.23 **	1					
4. Infrastructure	−0.34 **	−0.35 **	0.04	1				
5. Participation	−0.67 **	−0.63 **	0.01	0.42 **	1			
6. Planning	−0.42 **	−0.40 **	0.08	0.55 **	0.71 **	1		
7. Close relation	−0.13 **	−0.08 **	0.07	0.28 *	0.28 *	0.27 *	1	
8. Conflict	−0.35 **	−0.34 **	−0.12	0.17 *	0.15 *	0.14 *	−0.24 **	1
9. Dependency	−0.35 **	−0.33 **	0.03	0.29 **	0.35 **	0.22 **	0.68 **	0.19 *

Notes: * *p* < 0.05, ** *p*< 0.001.

## Data Availability

Dataset available on request from the authors.
